# Publication of Randomized Clinical Trials in Pediatric Research: A Follow-up Study

**DOI:** 10.1001/jamanetworkopen.2018.0156

**Published:** 2018-05-18

**Authors:** Leah K. Crockett, George N. Okoli, Christine J. Neilson, Rasheda Rabbani, Ahmed M. Abou-Setta, Terry P. Klassen

**Affiliations:** 1 George and Fay Yee Centre for Healthcare Innovation, Winnipeg, Manitoba, Canada; 2 Department of Community Health Sciences, Max Rady College of Medicine, Rady Faculty of Health Sciences, University of Manitoba, Winnipeg, Manitoba, Canada; 3 Neil John Maclean Health Sciences Library, University of Manitoba, Winnipeg, Manitoba, Canada; 4 Department of Pediatrics and Child Health, Max Rady Faculty of Health Sciences, University of Manitoba, Winnipeg, Manitoba, Canada; 5 Children’s Hospital Research Institute of Manitoba, Winnipeg, Manitoba, Canada

## Abstract

**Importance:**

Nonpublication of research results in considerable research waste and compromises medical evidence and the safety of interventions in child health.

**Objective:**

To replicate, compare, and contrast the findings of a study conducted 15 years ago to determine the impact of ethical, editorial, and legislative mandates to register and publish findings.

**Design, Setting, and Participants:**

In this cohort study, abstracts accepted to the Pediatric Academic Societies (PAS) meetings from May 2008 to May 2011 were screened in duplicate to identify phase 3 randomized clinical trials enrolling pediatric populations. Subsequent publication was ascertained through a search of electronic databases in 2017. Study internal validity was measured using the Cochrane Risk of Bias Tool, the Jadad scale, and allocation concealment, and key variables (eg, trial design and study stage) were extracted. Associations between variables and publication status, time to publication, and publication bias were examined.

**Main Outcomes and Measures:**

Publication rate, trial registration rate, study quality, and risk of bias.

**Results:**

A total of 177 787 abstracts were indexed in the PAS database. Of these, 3132 were clinical trials, and 129 met eligibility criteria. Of these, 93 (72.1%; 95% CI, 53.8%-79.1%) were published. Compared with the previous analysis, the current analysis showed that fewer studies remained unpublished (183 of 447 [40.9%] vs 36 of 129 [27.9%], respectively; odds ratio [OR], 0.56; 95% CI, 0.36-0.86; *P* = .008). Fifty-one of 129 abstracts (39.5%) were never registered. Abstracts with larger sample sizes (OR, 1.92; 95% CI, 1.15-3.18; *P* = .01) and those registered in ClinicalTrials.gov (OR, 13.54; 95% CI, 4.78-38.46; *P* < .001) were more likely to be published. There were no differences in quality measures, risk of bias, or preference for positive results (OR, 1.60; 95% CI, 0.58-4.38; *P* = .34) between published and unpublished studies. Mean (SE) time to publication following study presentation was 26.48 (1.97) months and did not differ between studies with significant and nonsignificant findings (25.61 vs 26.86 months; *P* = .93). Asymmetric distribution in the funnel plot (Egger regression, −2.77; *P* = .04) suggests reduced but ongoing publication bias among published studies. Overall, we observed a reduction in publication bias and in preference for positive findings and an increase in study size and publication rates over time.

**Conclusions and Relevance:**

These results suggest a promising trend toward a reduction in publication bias and nonpublication rates over time and positive impacts of trial registration. Further efforts are needed to ensure the entirety of evidence can be accessed when assessing treatment effectiveness.

## Key Points

**Question:**

What proportion of abstracts submitted to a major annual pediatric scientific meeting are subsequently published?

**Findings:**

Among 129 phase 3 randomized clinical trials identified in this cohort study, 27.9% were not subsequently published, and 39.5% were never registered, with previous trial registration and sample size associated with greater likelihood of publication. Mean (SE) time to publication from study presentation was 26.48 (1.97) months, and there was evidence of publication bias among published studies.

**Meaning:**

Further encouragement and follow-up are needed to ensure that the totality of evidence is made available to inform clinical decision making.

## Teaser

This study describes the proportion of clinical trials presented as abstracts at an annual pediatric scientific meeting that were ultimately published in the medical literature, and trial characteristics associated with publication vs nonpublication.

## Introduction

Publication bias, the selective publication of results or studies favoring positive outcomes, presents a critical threat to the validity and effectiveness of evidence-based medicine.^1^ First referred to as the “file-drawer problem” nearly 4 decades ago,^2^ selective publication has significant economic impacts, affects the safety of drugs and therapies, and stifles the implementation of true evidence-based medicine. This includes exposing study participants to potential harm due to duplication of previously unreported studies that were unsuccessful and creating a skewed evidence base for clinical decision making^3,4,5^ and knowledge translation efforts. The existence of publication bias may be even more problematic in the pediatric population because of the unique challenges of conducting controlled clinical trials in children, including small sample sizes, editorial bias of pediatric trials, hesitation to test interventions in children, challenges with informed consent, and limited funding allotted to pediatric research.^6^

Randomized clinical trials (RCTs) involve the enrollment of human participants to test a new treatment or intervention and provide the highest level of evidence to inform clinical practice.^7^ Studies at the phase 3 stage are typically designed to assess the efficacy of an intervention and its value in clinical practice and are generally the most expensive, time-consuming, and difficult trials to conduct, as they enroll a large number of patients.^7^ Similarly, studies at earlier stages are essential to assess safety and efficacy and to inform larger phase 3 trials.^7^ Thus, nonpublication at any stage of pediatric clinical trials has a direct impact on patients’ and populations’ health, represents a waste of human and financial resources, and violates our ethical imperative to share results and reduce harm, particularly among vulnerable segments of the population.^8,9,10^

A 2002 study examining abstracts accepted to the annual Pediatric Academic Societies (PAS) meetings between 1992 and 1995 and following up until 2000 to determine which studies were published found that 40.9% of abstracts reporting phase 3 RCTs with pediatric outcomes went unpublished, and that those with negative findings were less likely to be published.^11^ Since this study’s publication, a number of strategies have been implemented to minimize publication bias and increase transparency in clinical research,^9,12^ in particular the refusal by major journals to publish studies without prior trial registration.^13^ Despite these enhanced editorial mandates, publication bias persists.^14,15,16,17,18^ The purpose of this study was to replicate the methods of the previous study by (1) measuring the subsequent publication of abstracts presented at a major annual pediatric scientific meeting, (2) investigating trial quality and factors associated with publication, and (3) examining the association of trial registration with subsequent publication.

## Methods

### Selection of Abstracts and Articles

Conference abstracts presented at the PAS meetings between May 2008 and May 2011 and their identifying information were extracted from the PAS website. In collaboration with a knowledge synthesis librarian, a modified version of the Cochrane Highly Sensitive Search Strategy^19^ was developed and implemented to identify controlled clinical trials. Titles and abstracts of identified citations were then screened in duplicate using prespecified criteria to identify phase 3 RCTs that enrolled the pediatric population (neonatal, infants, children, and adolescents) and reported on pediatric outcomes. Studies reporting outcomes on pregnant women, animal studies, and non-RCT studies were excluded. No restrictions were imposed on treatment duration. Between March 20, 2017, and April 20, 2017, a search for trial registry results and full-text peer-reviewed publications corresponding to the citations that met the broad inclusion criteria was conducted in ClinicalTrials.gov and PubMed. A secondary search was then conducted in Embase, Web of Science, and Scopus for full-text articles not identified in PubMed. At least 1 of the a priori established primary outcomes must have been identified in the abstract and article to be considered as the corresponding article. The full-text search was conducted by a single researcher, with a second and third researcher conducting random checks for quality assurance. Ethical review and informed consent were not required by the University of Manitoba Health Research Ethics Board, as this was a review of a publicly available database and its associated articles in which aggregated data could not be associated with any individuals.

### Assessment of Methodological Quality

Internal validity of abstracts and articles was assessed using multiple methods. For consistency with the previous study, study quality was first scored using the validated 5-point Jadad scale,^20^ assessing randomization method, blinding, and description of dropouts and withdrawals. Second, study risk of bias was assessed as high, low, or unclear using the Cochrane Risk of Bias Tool^21^ according to 6 domains: sequence generation, allocation concealment, blinding, incomplete outcome data, selective outcome reporting, and other sources of bias; a final overall assessment within or across studies was then based on the responses to individual domains.^21^ Third, the method used to prevent foreknowledge of group assignment by patients and investigators was assessed. Allocation concealment was rated as adequate (eg, centralized or pharmacy-controlled randomization), inadequate (eg, any procedure that was transparent before allocation, such as an open list of random numbers), or unclear. It should be noted that allocation concealment is also assessed as part of the risk of bias assessment. Finally, any reported funding source was documented as government, pharmaceutical, private, other, or unclear.

### Data Extraction

Information extracted from identified abstracts and articles included abstract and journal citation, year of publication, trial design (parallel or crossover), study type (efficacy or equivalency), study stage (interim or final analyses), pilot or full trial, and primary study outcomes. Study type was noted based on the authors’ primary hypothesis: intent to demonstrate a significant difference between treatments (efficacy) or intent to show noninferiority between treatments (equivalence). A study was noted as efficacious (favoring the intervention) based on the authors’ statements and primary outcome significance level.

### Statistical Analysis

As per the previous study, medians and interquartile ranges were used to describe nonparametric data, means and standard deviations were used for normal continuous data, and percentages and 95% confidence intervals were used for dichotomous and categorical data. Pearson χ^2^ tests (dichotomous and categorical data), *t* tests (normal continuous data), and Wilcoxon rank sum tests (nonparametric data) were used to examine association between variables and publication status. Variables associated with publication were assessed using logistic regression, time to publication was assessed using log-rank tests, and publication bias was assessed using a funnel plot and Egger regression. Statistical significance was set at *P* < .05 (2-tailed test).

## Results

### Sample

We identified 177 787 abstracts indexed in the PAS database for the annual PAS conference from 2008 to 2011. Using a modified version of the Cochrane Highly Sensitive Search Strategy, we selected 3132 citations of controlled clinical trials for further review. Following title and abstract screening by 2 independent researchers, 129 citations met the eligibility criteria. Of these, 93 (72.1%; 95% CI, 53.8%-79.1%) were found as published articles. Table 1 summarizes the number of abstracts identified per year, their subsequent publication rate, and trial registrations in ClinicalTrials.gov per year.

**Table 1.** Trial Registration and Publication Status by Year

Year	Total Abstracts, No.	Abstracts, No. (%)		Published Articles, No. (%)	
		Published	Registered	Registered Abstracts (n = 78)	Unregistered Abstracts (n = 51)
2008	28	23 (82.1)	18 (64.3)	17 (94.4)	6 (60.0)
2009	29	20 (69.0)	19 (65.5)	16 (84.2)	4 (36.6)
2010	36	24 (66.7)	17 (47.2)	15 (88.2)	9 (47.4)
2011	36	26 (72.2)	24 (66.7)	23 (95.8)	3 (27.3)
Total	129	93 (72.1)	78 (60.5)	71 (91.0)	22 (43.1)

Overall, 78 abstracts (60.5%; 95% CI, 51.9%-68.5%) were registered in ClinicalTrials.gov. Fifty-one abstracts (39.5%; 95% CI, 31.5%-48.2%) were never registered. Of the registered abstracts, 71 (91.0%; 95% CI, 81.8%-96.0%) were subsequently published in peer-reviewed journals. Unregistered (n = 51) abstracts were significantly more likely to go unpublished (odds ratio [OR], 13.54; 95% CI, 4.78-38.46; *P* < .001). Articles were published in 53 journals: 12 articles (12.9%) in *Pediatrics*, 8 (8.6%) in *Journal of Pediatrics*, 4 (4.3%) in *Pediatric Emergency Care*, and the remaining with 3 or fewer (<3.5%) in any 1 journal. Articles were published in 28 specialist pediatric journals and 26 not specific to pediatrics. Median (range) impact factor was 3.223 (0.945-72.406).

Overall, there were few abstracts reporting pilot studies (8 abstracts [6.2% overall; 2.8% of unpublished abstracts and 7.5% of published abstracts]) or studies in preliminary stages (7 abstracts [5.4% overall; 0% of unpublished studies and 7.5% of published studies]). Study withdrawals, defined as participants leaving the study either voluntarily or when asked to by the investigators, were reported in only 16 abstracts (12.4% overall; 11.1% of unpublished abstracts and 12.5% of published abstracts).

### Quality of Abstracts and Articles

Quality measures for abstracts and articles are summarized in Table 2. There were no distinguishable differences in quality between abstracts that were subsequently published and those that were not with the exception of an assessment of high risk of bias, which was more easily distinguishable in unpublished vs published abstracts. Because most abstracts did not contain sufficient information to score quality measures, full manuscript text was examined to generate the Jadad and Risk of Bias scores for published and unpublished articles and identify allocation concealment and funding source for published articles.

**Table 2.** Quality Variables by Document Type

Quality Measure	Abstracts		Articles (n = 93)
	Not Subsequently Published (n = 36)	Subsequently Published (n = 93)	
Jadad score, median (IQR)	1 (1-2)	1 (1-2)	3 (2-5)
Risk of bias score, % (95% CI)			
Low	0	0	26.9 (18.9-36.7)
Unclear	77.8 (61.9-88.3)	89.2 (81.3-94.1)	41.9 (32.4-52.1)
High	22.2 (11.7-38.1)	10.8 (5.9-19.3)	31.2 (22.7-41.2)
Allocation concealment, % (95% CI)			
Adequate	0	0	62.4 (52.2-71.5)
Unclear	100	100	1.1 (0.2-5.9)
Inadequate	0	0	36.5 (27.5-46.7)
Funding source, % (95% CI)			
Government	0	5.4 (2.3-12.0)	24.8 (17.4-33.9)
Pharmacy	0	15.1 (9.2-23.7)	28.7 (20.8-38.2)
Private	0	4.3 (1.7-10.5)	18.8 (12.4-27.5)
Unclear	100	24.7 (17.1-34.4)	27.7 (19.9-37.1)

Abbreviation: IQR, interquartile range.

### Variables Associated With Publication

Variables associated with publication based on the univariate analysis are summarized in Table 3. Trial registration was significantly associated with study publication compared with no study publication (76.3% [95% CI, 66.8%-83.8%] vs 19.4% [95% CI, 97.5%-35.0%], respectively; *P* < .001), as was sample size (median [interquartile range] sample size, 133 [57-312] vs 67 [47-126], respectively; *P* = .01). All other variables remained insignificant. Similarly, in the adjusted analysis, sample size (OR, 1.92; 95% CI, 1.15-3.18; *P* = .01) and trial registration (OR, 13.54; 95% CI, 4.78-38.46; *P* < .001) were associated with subsequent publication (Table 4).

**Table 3.** Univariate Results for Variables Associated With Publication

Variable	Abstracts, No. (%)		OR (95% CI)
	Not Subsequently Published (n = 36)	Subsequently Published (n = 93)	
Dichotomous			
Overall study conclusions favoring treatment	16 (44.4)	50 (53.8)	1.43 (0.67-3.15)
Trial registered in ClinicalTrials.gov (vs unregistered)	7 (19.4)	71 (76.3)	13.37 (5.15-34.71)
Equivalency studies (vs efficacy)	7 (19.4)	21 (22.6)	1.21 (0.46-3.15)
Parallel study design (vs crossover)	34 (94.4)	85 (91.4)	0.63 (0.13-3.09)
Categorical			
Year			
2008	5 (13.9)	23 (24.7)	2.04 (0.71-5.85)
2009	9 (25.0)	20 (21.5)	0.82 (0.33-2.03)
2010	12 (33.3)	24 (25.8)	0.70 (0.30-1.60)
2011	10 (27.8)	26 (28.0)	1.01 (0.43-2.38)
Discrete nonparametric			
Sample size, median (IQR), No.	67 (47-126)	133 (57-312)	NA

Abbreviations: IQR, interquartile range; NA, not applicable; OR, odds ratio.

**Table 4.** Variables Associated With Publication of 127 Results From Logistic Regression

Variable	Odds Ratio (95% CI)	*P* Value
Efficacy study (vs equivalence)	0.88 (0.26-3.01)	.83
Significant findings (vs not)	1.60 (0.58-4.38)	.36
Sample size (for every log-log unit increase)	1.92 (1.15-3.18)	.01
Parallel design (vs crossover)	1.04 (0.15-7.18)	.97
Full study (vs pilot)	1.13 (0.60-2.98)	.07
Registered (vs not)	13.54 (4.78-38.46)	<.001

### Variables That Changed From Abstract to Published Article

Ninety-three abstracts were paired with their subsequently published articles. Of these, 50 (53.8%; 95% CI, 43.7%-63.5%) had conclusions favoring treatment, which was similar to abstracts that went unpublished (44.4%; 95% CI, 29.5%-60.4%; *P* = .34). The effect size reported in 26 of 93 published articles (28%) differed from that reported in the abstracts; 6 (6.5%) changed from favoring the treatment in the abstract to not favoring the treatment in the article, and 3 (3.2%) changed from not favoring treatment in the abstract to favoring the treatment in the article. In other studies, while overall conclusions did not change, 11 studies (11.8%) increased in effect size from abstract to article, 8 (8.6%) decreased in effect size, and sample size increased from abstract to article in 6 studies (6.5%).

### Time to Publication

The time to publication by overall study conclusions (favoring treatment or not) was assessed. Favoring the new therapy had no association with time to publication (25.61 [95% CI, 25.28-25.94] vs 26.86 [95% CI, 26.58-27.14] months; *P* = .93). Overall, the mean (SE) time to publication following presentation at PAS was 26.48 (1.97) months. Median time to publication was 24 months (95% CI, 19.0-27.0 months), with lower and upper quartile estimates ranging from 12 months (95% CI, 9.0-17.0 months) to 35 months (95% CI, 31.0-41.0 months), respectively.

### Publication Bias

Examination of funnel plot symmetry is presented in the Figure, depicting effect estimates from individual studies against study precision for studies presenting categorical data. Overall, the funnel plot suggests no presence of publication bias (Egger regression, −1.19; *P* = .20). The funnel plot with published studies only shows asymmetric distribution (Egger regression, −2.77; *P* = .04). However, no asymmetry was observed in unpublished studies (Egger regression, 0.52; *P* = .70). The asymmetry of the plot suggests that studies favoring the control group are absent among published studies, and it may be due to nonsubmission of studies favoring controls to scientific meetings (submission bias).

**Figure.**
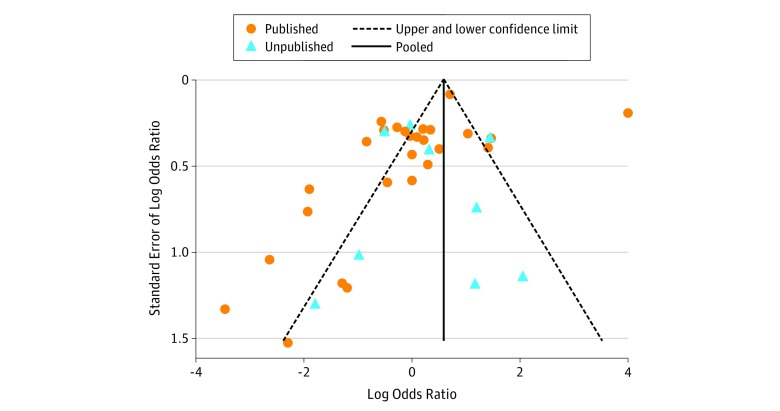
Funnel Plot of Standard Error by Log Odds Ratio The plot shows pseudo–95% confidence limits of published and unpublished studies. Egger regression: published bias = −2.77; *P* = .04 vs unpublished bias = 0.52; *P* = .70. Overall bias = −1.19; *P* = .20.

### Comparison With the 2002 Sample

Fewer articles remained unpublished since the 2002 study (36 of 129 [27.9%] vs 183 of 447 [40.9%]; OR, 0.56; 95% CI, 0.36-0.86; *P* = .008), and preference for positive results was no longer significant (OR, 2.24; 95% CI, 1.51-3.34; *P* < .001 vs OR, 1.60; 95% CI, 0.58-4.38; *P* = .34). Although publication bias was still present in the subgroup analysis of published studies, overall, publication bias was absent, study quality was higher, and sample sizes were larger.

## Discussion

The results of this study suggest a promising trend in the reduction of nonpublication and positive impacts of trial registration on subsequent publication. However, while fewer manuscripts remain unpublished since the 2002 study (27.9% vs 40.9%; OR, 0.56; 95% CI, 0.36-0.86; *P* = .008), nonpublication of results from phase 3 pediatric RCTs remains high with nearly 3 of every 10 studies placed in the file drawer. In this sample of unpublished studies, this represents 4192 children directly exposed to interventions that did not lead to informative findings, and potentially more who may have been indirectly affected because of duplication of previously unreported studies and/or clinical decisions made based on an imprecise evidence base. This suggests that further safeguards are needed for patients and families to ensure that they are not entered into trials that are underpowered, methodologically poor, wasteful of scarce research resources, and ultimately unlikely to be published; however, we are seeing positive steps forward in this regard.

In this sample, most abstracts were not detailed enough to adequately rate study quality and most studies reporting funding sources at the abstract stage were funded by the pharmaceutical industry. At the abstract stage, most studies were rated of low quality based on the Jadad scale or had an unclear risk of bias; this did not differ between published and unpublished studies. However, the use of these tools for quality assessment was intended for articles, with limited space to adequately detail required quality measures in abstracts, and as such, articles were rated of high quality at publication stage. Furthermore, we observed an increase in quality scores from the previous sample. Mean (SE) time to publication following presentation at the PAS meeting was 26.48 (1.97) months, with 23 of 93 authors (25%) publishing study findings within 1 year.

A major finding from this study is the impact of trial registration and the refusal of major journals to publish the findings of unregistered trials. Our analysis found trial registration in ClinicalTrials.gov to be significantly associated with publication, increasing the odds of subsequent publication by 13.5 times (*P* < .001). Despite this finding and important steps and regulations to produce more comprehensive registers, only 60.5% of trials (78 of 129) in this sample were registered. Although the importance of prospective trial registry is widely recognized,^22,23^ similar rates of nonadherence have been reported elsewhere.^15,17,24^ Even though trial registration was associated with increased odds of subsequent publication, a proportion of registered trials remain unpublished. This is consistent with previous research suggesting that mandatory trial registration alone is not sufficient to reduce publication bias and that a multifaceted approach is needed to enhance mandatory transparency of research with human participants.^22,25,26^ Funders, journals, and ethics committees could have an important role to play in encouraging stronger enforcement of trial registration. Further expansion of this policy should include a contingency by ethics boards to only award ethics approval on proof of trial registration to ensure that no studies commence prior to trial registration. Methods to reduce publication bias have been explored previously, and research registration was viewed as the most effective method by journal editors.^27^ However, enforcement of registration was viewed less favorably by researchers and academics.^27^

Unlike the previous study, study significance was not found to be associated with subsequent publication among abstracts presented between 2008 and 2011 at the PAS scientific meeting. However, it is unclear how many RCTs performed in children were never submitted or accepted to an annual meeting. Our study found an overall trend of publication bias among published studies. However, this was analyzed only on a portion of all included studies and, as such, should be interpreted with caution. Furthermore, the overall evidence for publication bias in this analysis is modest.

Nonpublication of research findings has implications. Failure to publish results of clinical research involving participants and families who have consented to participate disrespects their personal contribution and decreases public trust in clinical science.^28^ The persistence of nonpublication of trial findings nearly 15 years later^11^ violates the Declaration of Helsinki, which states that every research study involving human participants must be registered in a publicly accessible database before the recruitment of the first participant and that researchers, authors, editors, and publishers have an ethical obligation to publish research results.^9^ Timely publication of the findings of clinical trials is essential not only to support evidence-based decision making by patients and clinicians, but also to ensure that all children receive the best possible care.

### Limitations

Several limitations should be noted. Although the PAS meetings serve as a useful sample with which to examine publication bias and nonpublication of research findings, they do not capture the full breadth of existing trials. Recent studies have also examined the discontinuation and nonpublication of RCTs conducted in children based on trials registered in ClinicalTrials.gov.^15^ Our study was able to capture additional trials that were never registered (51 of 129 [39.5%]) and demonstrates that trial registration, although associated with subsequent publication, does not guarantee publication. Furthermore, our findings suggest that a significant proportion of trials remain unregistered. This is an important addition to the literature. Second, our replication study yielded a lower sample of phase 3 RCTs compared with the previous study.^11^ However, predefined inclusion criteria were followed by 2 independent reviewers. Furthermore, exact definitions used in the previous study were used at the stage of variable extraction. Variation in sample size between the 2 studies could be due to more stringent inclusion criteria or a shift in the research realm or conference interests over time. Finally, because of limited space within abstracts, it is difficult to judge study quality at this stage. However, quality did not differ between abstracts that were subsequently published and those that were not.

## Conclusions

Our study found that among phase 3 pediatric RCTs presented at a major scientific meeting, a large proportion of studies remain unregistered and unpublished. However, we observed promising trends in the reduction of nonpublication and positive impact of editorial policy changes. Of studies that were published, previous trial registration and larger sample sizes were significantly associated with subsequent publication. These findings suggest that ongoing efforts are needed to enhance publication of all trials and to enforce mandatory trial registration, particularly in pediatric trials, to ensure that participation and knowledge generation can benefit all children.
